# Robust, High-Performing Maize–Perovskite-Based Solar Cells with Improved Stability

**DOI:** 10.1021/acsaem.1c02058

**Published:** 2021-09-27

**Authors:** Antonella Giuri, Nicholas Rolston, Silvia Colella, Andrea Listorti, Carola Esposito Corcione, Hannah Elmaraghi, Simone Lauciello, Reinhold H. Dauskardt, Aurora Rizzo

**Affiliations:** † CNR NANOTEC, Institute of Nanotechnology, Via Monteroni, Lecce 73100, Italy; ‡ Department of Materials Science and Engineering, Stanford University, Stanford, California 94305, United States; § CNR NANOTEC—Istituto di Nanotecnologia, Dipartimento di Chimica, Università degli Studi di Bari Aldo Moro, Via Orabona 4, Bari 70126, Italy; ∥ Dipartimento di Chimica, Università degli Studi di Bari Aldo Moro, Via Orabona 4, Bari 70126, Italy; ⊥ Dipartimento di Ingegneria dell’Innovazione, Università del Salento, via per Monteroni, km 1, Lecce 73100, Italy; # Electron Microscopy Facility, Istituto Italiano di Tecnologia, via Morego 30, Genova 16163, Italia

**Keywords:** perovskite solar cell, starch, mechanical reinforcement, grain boundaries, thermomechanical stability, photostability

## Abstract

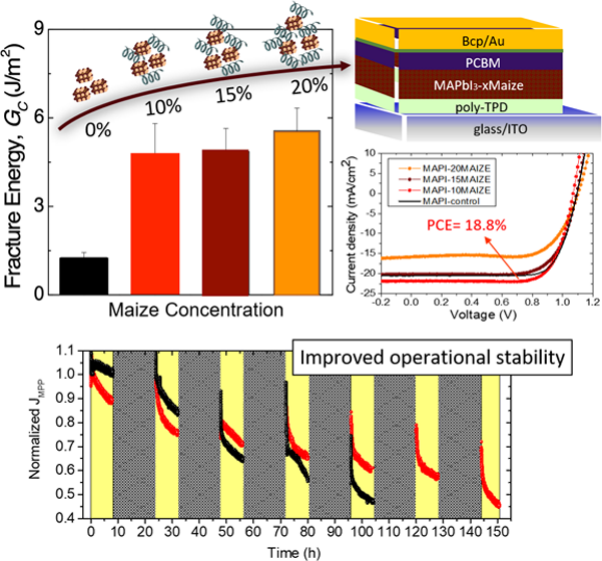

Herein, we focus on improving the long-term chemical and thermomechanical stability of perovskite solar cells (PSCs), two major challenges currently limiting their commercial deployment. Our strategy incorporates a long-chain starch polymer into the perovskite precursor. The starch polymer confers multiple beneficial effects by forming hydrogen bonds with the methylammonium iodide precursor, templating perovskite growth that results in a compact and homogeneous film deposited in a simple one-step coating (antisolvent-free). The inclusion of starch in the methylammonium lead iodide films strongly improves their thermomechanical and environmental stability while maintaining a high photovoltaic performance. The fracture energy (*G*_c_) of the film is increased to above 5 J/m^2^ by creating a nanocomposite that provides intrinsic reinforcement at grain boundaries. Additionally, improved optoelectronic properties achieved with the starch polymer enable good photostability of the active layer and enhanced resistance to thermal cycling.

## 1 Introduction

The outstanding optoelectronic properties^1−4^ make hybrid halide perovskites ideal photoactive materials for next-generation solar cells, enabling the development of single-junction lab-scale devices with a record power conversion efficiency (PCE) of up to 25.5% only 12 years after the first demonstration of perovskite solar cells (PSCs).^5,6^ However, significant perovskite compositional and structural degradation mechanisms result from the intrinsic thermal and chemical instabilities in the presence of several factors, such as oxygen and humidity under ambient conditions, at visible and UV light, high temperature, and electrical bias.^7−13^ Furthermore, perovskites are mechanically fragile and require improvement to produce a durable material compatible with large-area manufacturing.^14^ The biggest challenges currently limiting the commercial deployment and manufacturability of PSCs are the lack of long-term chemical and thermomechanical stability.^15−17^

Improving the durability of perovskite materials under operational conditions remains a requirement for device scalability. In addition to inherent mechanical fragility, the stresses generated in perovskite films during fabrication create a driving force for defect generation and propagation during operation, contributing significantly to light-, heat-, and moisture-based chemical degradation as well as fracture in devices.^18^ The fracture energy, *G*_c_, represents one of the most important thermomechanical reliability metrics of PSCs.^19^ Previous work for a range of monolithic PSCs featuring diverse architectures, cation compositions, and microstructures^14,20^ discovered that the *G*_c_ of perovskite films (<1.5 J/m2) was the lowest of any photovoltaic technology. As such, an increased resistance to fracture is central to improve the operational stability and manufacturability of PSCs.

Various strategies have been developed to improve perovskite thermomechanical stability: (i) intrinsically, through the engineering of the material by tuning the cation composition or introducing bulky cation additives that modify the perovskite crystal structure to form 2D perovskite layers or by adding additives that stabilize and protect perovskite from decomposition^21−26^ and (ii) extrinsically, involving the use of scaffold-based reinforcement—although these efforts introduce an additional processing step during fabrication^27,28^—or to a lesser extent, device encapsulation with different packaging materials.^20^

In previous work, an effective intrinsic toughening approach incorporating a long-chained amino acid additive, 5-aminovaleric acid (5-AVA), was proposed—showing a significant improvement in the *G*_c_ of perovskite—but at the expense of reduced charge extraction and device performance with the addition of a bulky, insulating organic layer.^29^ Our recently developed perovskite–starch-based nanocomposite^23^ showed improved tolerance to moisture when incorporated into PSCs at optimized concentrations without the penalty of reducing the device performance. We have demonstrated that the starch also modifies the rheological behavior of the perovskite precursor solution and interacts with methylammonium iodide (CH_3_NH_3_I; MAI) by hydrogen bonding, forming a persistent sol–gel network that delays the crystallization process.^30^ The longevity of the wet precursor solvate phase guarantees the formation of a compact and homogeneous film morphology in a single-step coating (antisolvent dripping-free) and enhances the thermodynamic stability of the starch–perovskite composite. Most importantly, the as-developed material used as the active layer of PSCs shows very high power conversion efficiency despite the insulating properties of the starch. Moreover, the hygroscopic nature of the long-chain starch network likely stabilizes and protects the perovskite from decomposition in an ambient environment, improving the solar cell device moisture resistance, while the plasticity of the polymer conferred enhanced resistance to bending stress. In this work, we perform an in-depth study on the nanocomposite perovskite/starch film through mechanical, chemical, and morphological characterization with the aim of rationalizing how the long-chain starch polymer influences both the thermomechanical and environmental stability of methylammonium lead iodide (MAPbI_3_) perovskite under accelerated aging conditions.

## 2 Results

Films based on several starch/precursor ratio wt % (0–10–15–20), reported in Table S1, were fabricated in a one-step spin-coating deposition by following a previously optimized recipe.^23^ Compared with previous work,^23^ here, a purer corn starch from maize (Maize, ∼73% amylopectin and 27% amylose, purchased from Sigma-Aldrich, characterized by the chemical structure reported in Figure S1) was utilized.

**Figure 1:**
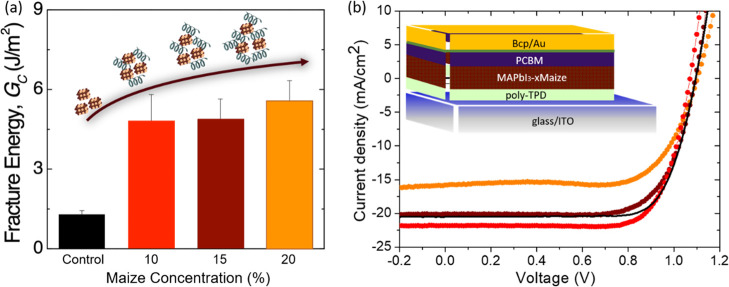
(a) *G*_c_ measured for different Maize concentrations (10–15–20 wt %) and MAPbI_3_-control with a sketch representing the increased Maize content in the inset. (b) Current–voltage characteristics for the best devices based on different Maize concentrations (10–15–20 wt %) and MAPbI_3_-control with a scheme of the photovoltaic device architecture based on the perovskite–Maize photoactive layer in the inset.

The influence of the polymer on the mechanical properties of perovskite was tested using the double cantilever beam (DCB) method, where perovskite films with varying concentration of Maize were deposited on poly(*N*,*N*′-bis-4-butylphenyl-*N*,*N*′-bisphenyl)benzidine (poly-TPD)/indium tin oxide (ITO)-glass substrates and bonded to an identical glass superstrate. The perovskite fracture energy (*G*_c_), reported in Figure 1a, was calculated from a load–displacement curve that was generated by applying a uniaxial load to one end of the beams. Once crack propagation occurred, the sample was unloaded slightly and reloaded to propagate the crack and enable several measurements of *G*_c_ across the length of the sample. MAPbI_3_ control films had a *G*_c_ value of 1.3 ± 0.2 J/m^2^, comparable to previous measurements on perovskite films without additives. The inclusion of Maize additives had a marked increase in *G*_c_ to 4.8 ± 1.0 J/m^2^ for 10 wt %, 4.9 ± 0.8 J/m^2^ for 15 wt %, and 5.6 ± 0.8 J/m^2^ for 20 wt %. A minor increase was observed with increased Maize concentration (from 10 wt % to 20 wt %), while the largest improvement was observed with the addition of 10 wt % Maize compared to control MAPbI_3_. This result indicates that starch effectively acts as an intrinsic reinforcement strategy that enhances perovskite *G*_c_ to exceed 5 J/m^2^, a value at which materials are considered mechanically robust and not susceptible to cracking from the stresses induced during processing and handling.^27^

Notably, the inclusion of Maize into perovskite showed very high photovoltaic performance, among the highest reported for fully organic p–i–n devices based on the MAPbI_3_–polymer active layer,^31^ especially considering that the nanocomposite films were produced with one-step spin coating without the use of an antisolvent or a curing source. In particular, the different concentrations of Maize into perovskite (from 10 to 20 wt %) were explored by implementing the films in a fully organic inverted device whose layout, reported in the inset of Figure 1b, comprised ITO/poly-TPD/MAPbI_3_–Maize/phenyl-C61-butyric acid methyl ester (PCBM)/bathocuproine (BCP)/gold (Au). As evidenced in Figure 1b and Table 1, the best photovoltaic performance was obtained with a MAPbI_3_-10Maize-based device, reaching a high PCE of 18.8% with a high open-circuit voltage (*V*_OC_) of 1.1 V. This excellent voltage output was likely due to the high-quality perovskite film obtained with the Maize additive that produced an optimized band energy alignment. Additionally, a high short-circuit current density (*J*_sc_) of 21.9 mA/cm^2^ and a fill factor (FF) of 78.3% suggested a low series resistance and reduced carrier recombination at the photoactive layer interfaces (confirming the results observed previously^23^ with a commercial starch). Importantly, these devices show no hysteresis when measured in forward and reverse scan directions, as reported in Figure S2.

**Table 1:** Photovoltaic Parameters of the Best Devices, Measured by Reverse Scans, and Average Reverse Values for the Different Maize Concentrations Explored (10–15–20 wt %) and MAPbI_3_-Control

		FF (%)	*V*_oc_ (V)	*J*_sc_ (mA/cm^2^)	PCE (%)
**MAPbI**_**3**_**-10Maize**	best device	**78.3**	**1.10**	**21.9**	**18.8**
	average	73.4 ± 5.5	1.09 ± 0.01	19.8 ± 1.6	15.7 ± 1.1
**MAPbI**_**3**_**-15Maize**	best device	**71.9**	**1.09**	**20.1**	**15.8**
	average	67.4 ± 6.4	1.08 ± 0.02	16.8 ± 1.7	12.3 ± 1.9
**MAPbI**_**3**_**-20Maize**	best device	**70.9**	**1.10**	**15.8**	**12.3**
	average	64.7 ± 2.8	1.11 ± 0.01	14.7 ± 1.4	10.6 ± 1.2
**MAPbI**_**3**_**-control**	best device	**75.5**	**1.09**	**20.5**	**17.0**
	average	72.0 ± 6.6	1.06 ± 0.05	19.8 ± 1.6	15.0 ± 1.6

Incident-photon-to-current efficiency (IPCE) spectra of the solar cells based on MAPbI_3_-10Maize, reported in Figure S3, showed a shape close to that of standard MAPbI_3_. By increasing the Maize content from 10 to 15 wt % the PCE decreased to 15.8% along with decreasing FF to 71.9%. The device based on the highest Maize content of 20 wt % showed a lower PCE of 12.3% with a decrease of both *J*_sc_ to 15.8 mA/cm^2^ and FF to 70.9%. By increasing the Maize content, the device performance decreased—in particular, the FF and *J*_sc_—likely due to the insulating nature of the polymer and due to the increase in the perovskite film thickness. Indeed, a noticeable increase in the thickness of the final film was observed from about 450 nm for MAPbI_3_-10Maize to 1 μm for MAPbI_3_-15Maize and up to 1.4 μm for MAPbI_3_-20Maize. The change in the film thickness with higher polymer content is primarily due to the increase in the viscosity of the perovskite precursor solution, as reported in previous work.^23^

**Figure 2:**
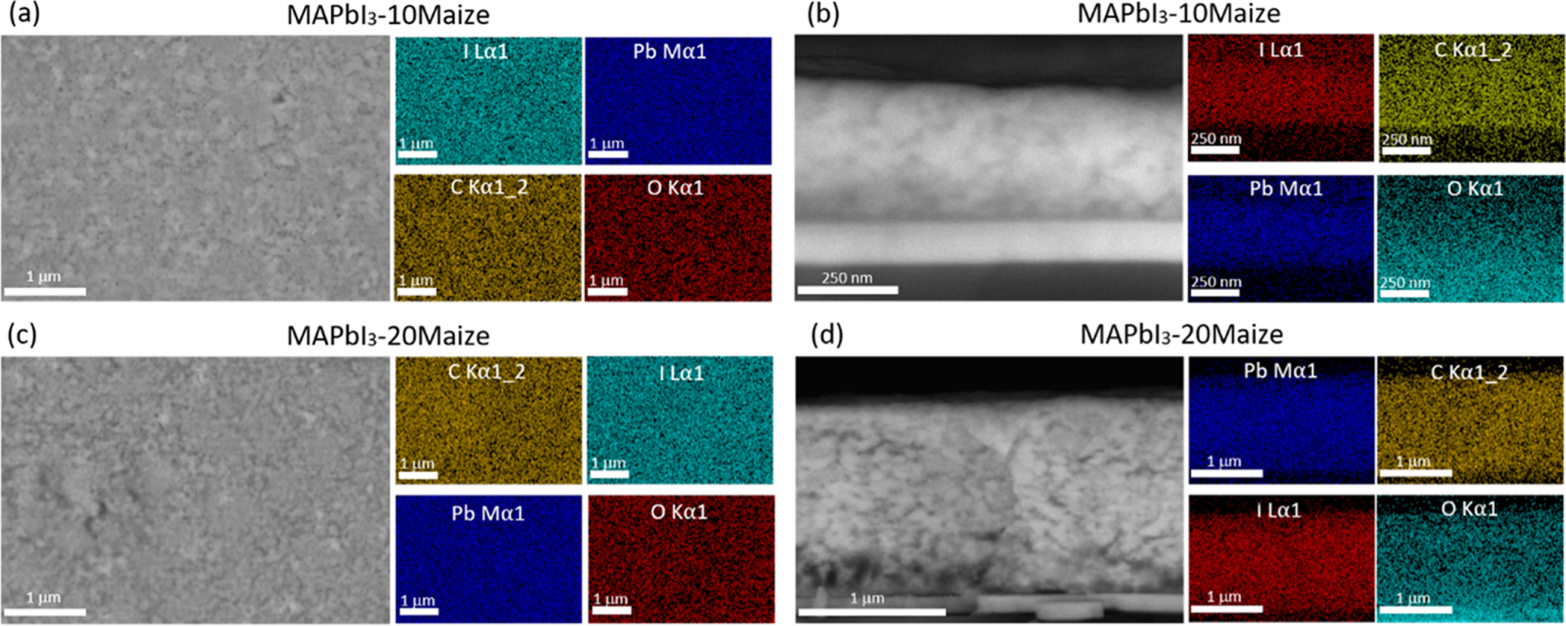
SEM–EDS images of MAPbI_3_-10Maize—top view (a) and cross section (b) and of MAPbI_3_-20Maize—top view (c) and cross section (d).

The morphological study of the influence of Maize inclusion into perovskite was carried out by scanning electron microscopy–energy dispersive X-ray spectroscopy (SEM–EDS) analyses reported in Figure 2. As evidenced from the top view of the films (Figure 2a,c), the inclusion of Maize enabled a smooth and homogeneous morphology of the perovskite layer, characterized by a uniform distribution of the polymer into the film, both on the top and through the thickness and around the grain boundaries. The presence of Maize throughout the thickness of the perovskite film indicates that the likely mechanism for improved *G*_c_ in the perovskite-Maize films is the reinforcement of grain boundaries, which have been found as weak points that enable crack propagation in typical, fine-grained MAPbI_3_ films.^32^ 10 wt % of Maize appears to be sufficient to reinforce the majority of grain boundaries within the film, as further increments of polymer addition only result in minor improvements in *G*_c_. Since the contributions to *G*_c_ are related to energy dissipated from bond breaking and from plastic deformation, the latter is potentially the cause of the slight increase from 10 to 20 wt % since the Maize is a compliant and plastically deformable polymeric material as compared to the brittle perovskite.

As can be observed from SEM–EDS analyses of the cross section of the film reported in Figure 2b,d, the polymer is homogeneously distributed along the thickness of the film, even at high concentrations (Figure 2d), resulting in the formation of a compact and uniform photoactive layer. However, it is interesting to note that despite the insulating nature of the polymer used, the device retains a respectable performance in the presence of high Maize content. This unexpected effect will be further explored in a later section by performing further characterization on the optoelectronic and chemical properties of the perovskite films.

**Figure 3:**
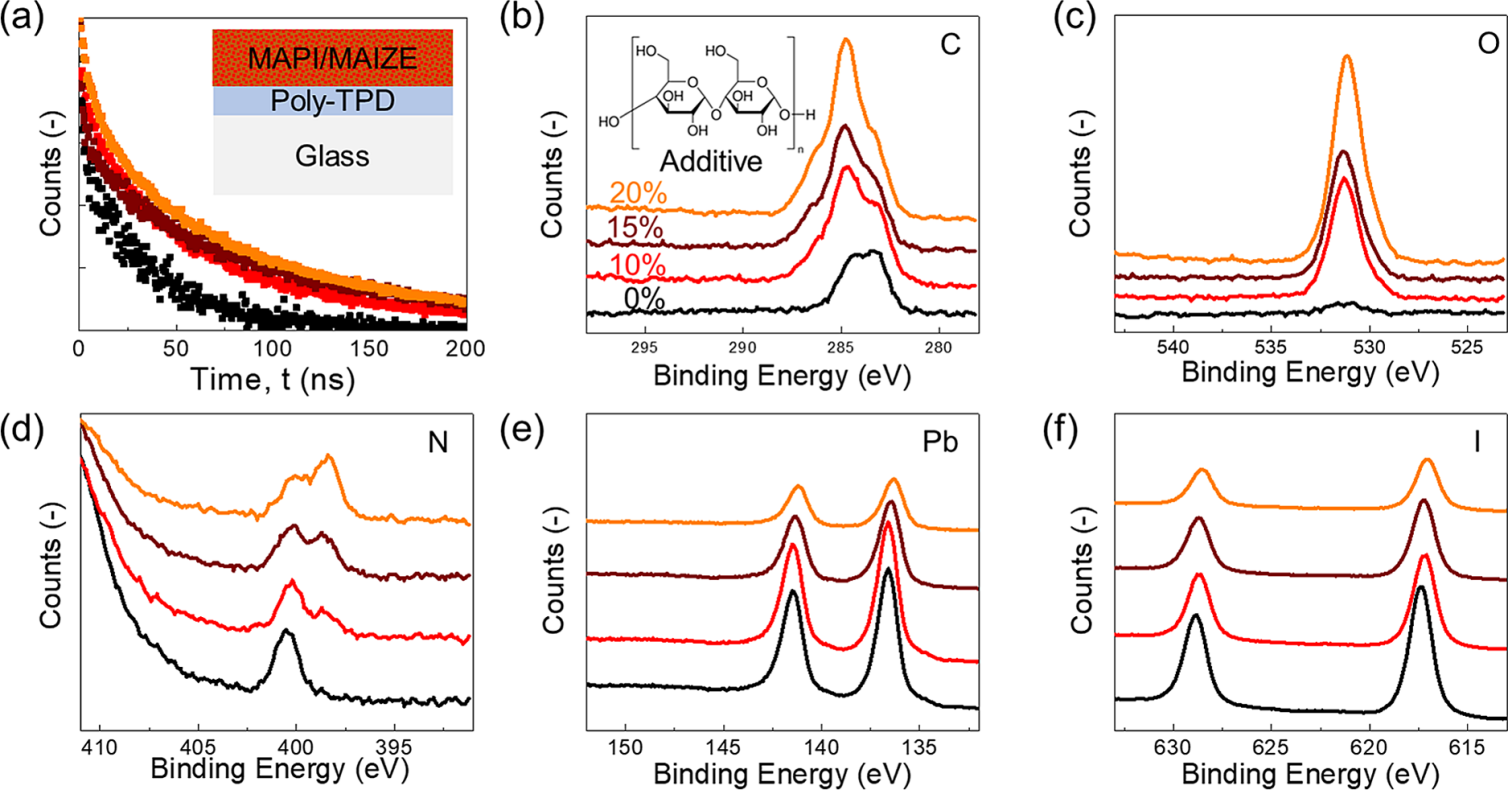
TRPL measurements of MAPbI_3_ (black), MAPI_3_-10Maize (red), MAPI_3_-15Maize (dark red), and MAPI_3_-20Maize (orange) (a); X-ray photoelectron spectroscopy (XPS) analysis of perovskite films with the same range of Maize concentrations tracking carbon (b), oxygen (c), nitrogen (d), lead (e), and iodine (f) peaks.

To further understand the mechanism for higher PCE and stability with the addition of Maize, time-resolved photoluminescence (TRPL) measurements were performed on glass/poly-TPD/perovskite films. A slight improvement in the charge carrier lifetime was observed for all Maize-containing perovskite compositions (Figure 3a), which indicates that surface recombination was likely mitigated by the polymer. The carrier lifetimes did not change markedly with increased Maize content, an effect that is similar to the measured *G*_c_ data. The observed correlation between mechanical and optoelectronic properties indicates that the changes in film chemistry and morphology induced by the polymer can improve both the device performance and thermomechanical reliability.

Larger carrier lifetime improvements have been observed in other studies with perovskite surface passivation strategies,^33,34^ but the minor improvements in lifetime with the addition of Maize coupled with the SEM–EDS maps indicate that Maize is uniformly distributed throughout the film rather than forming a continuous layer atop the perovskite film. This further supports the hypothesis that Maize acts as a rheology modifier that interacts with precursors and templates perovskite grain formation during the deposition, eventually segregating as a thin layer at the grain boundaries. This segregation is quite important to ensure adequate charge transport through the perovskite grains and is fundamental to the improved mechanical properties and stability.

The surface chemistry of the perovskite films in the presence and absence of Maize was characterized using XPS, and the results are reported in Figure 3b–f. The addition of Maize led to a noticeable increase in the oxygen 1s core level peak along with a concomitant reduction in the signal for the nitrogen 1s, lead 4f, and iodine 3d peaks. The higher Maize content enhanced the change in various peak intensities, which further supports the claim that Maize is not a surface passivating agent, but it thoroughly mixes with the perovskite domains in the active layer. In fact, higher Maize content leads to more additive-rich clusters throughout the thickness of the perovskite film and around grain boundaries. At 20 wt % Maize, the lead and iodine peaks are substantially reduced, and this large drop in the active elements of the perovskite could indicate one reason why *J*_sc_ was noticeably lower at this perovskite–Maize composition (Figure 1b), in addition to the previous observation that thicker films were produced at the highest Maize concentration. These characterization results indicate that controlling the Maize addition in the perovskite precursor can be used to effectively tune the film chemistry of the resulting perovskite–Maize nanocomposite film.

In our previous work, the stability of the developed perovskite–starch nanocomposite was analyzed by placing the films and the respective devices without encapsulation in ambient air (temperature: ∼22 °C; moisture content: 40–70%)^23^ to investigate the influence of the starch on moisture resistance. The observed retention of 50% of initial PCE after about 400 h of aging test, compared to the complete degradation of the control device just after 145 h, was attributed to the beneficial protection effect of the hygroscopic starch from ambient environment contamination (in particular, oxygen and moisture) according to the ISOS-D-1 protocol.^15^ Here, the device stability of the best performing composition, MAPbI_3_-10Maize, was investigated further through the use of separate accelerated aging protocols by evaluating the impact of thermal cycles (ISOS-T-1I) and the combination of light and bias in the presence of oxygen and moisture (ISOS-LC-1).^15^

**Figure 4:**
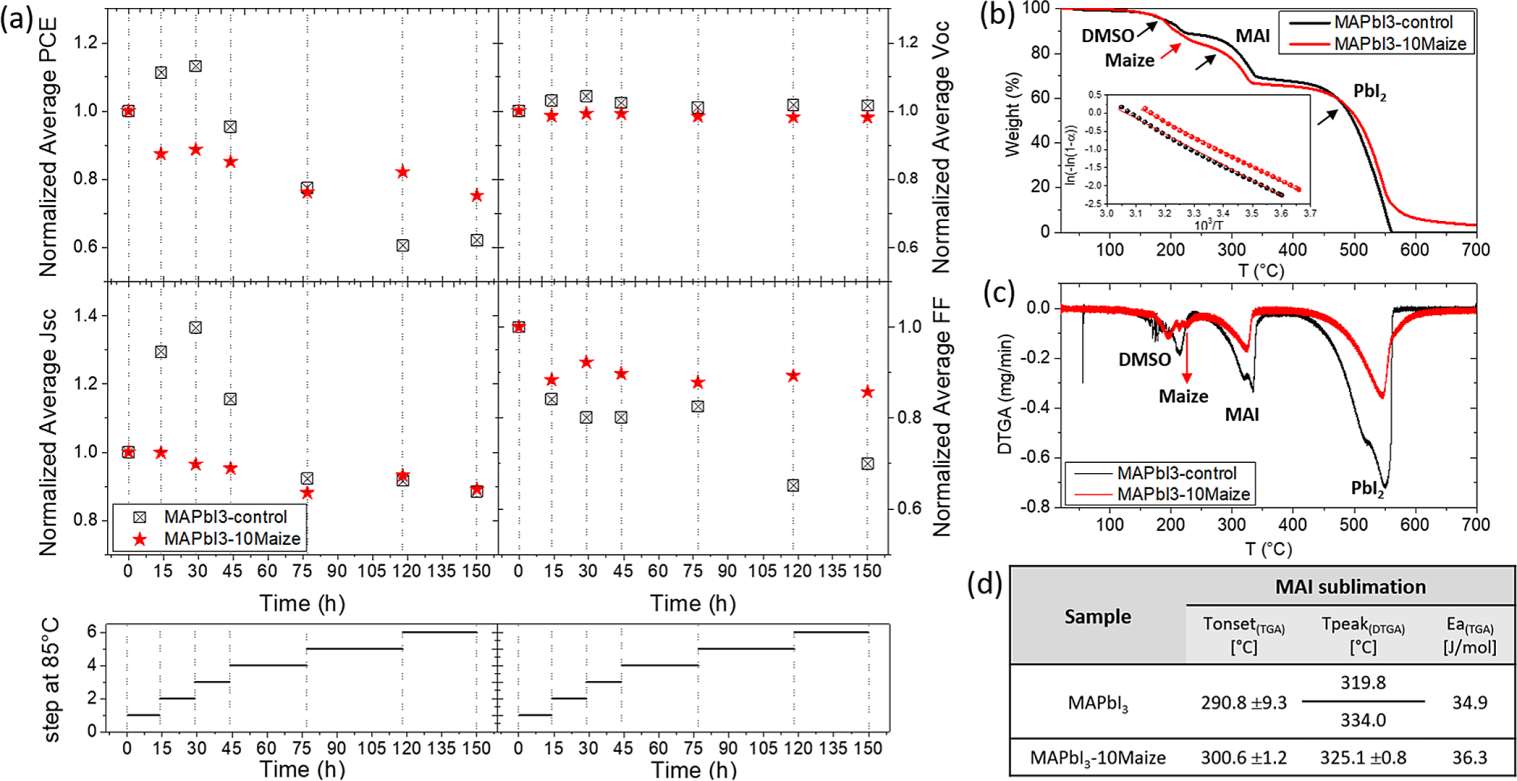
Normalized average parameters of PCE, *V*_OC_, *J*_SC_, and FF of MAPbI_3_-10Maize (red) and a reference MAPbI_3_ (black) perovskite (deposited by the dripping method)-based device as a function of thermal cycles, measured under the ISOS-T-1I-like aging condition (a); TGA (b) and DTGA (c) curves of perovskite material without and with Maize; linear fit of the data (based on eq 1) is displayed in the inset of figure (b); and characteristic parameters, extrapolated from TGA, related to MAI sublimation of perovskite material without and with Maize (d).

Thermal stability was investigated through device thermal cycling from room temperature to 85 °C under a nitrogen atmosphere, mimicking the temperature change during the day. As evidenced in Figure 4a, the degradation occurring in both devices was mainly due to a decrease in the FF, reducing the PCE by about 33% in MAPbI_3_-10Maize and 40% in standard MAPbI_3_ after six steps of cycling thermal aging for a total of 150 h of testing in temperature, as reported in Figure 4a, characterized by 8 h at room temperature between each step at high temperatures. In the device, thermal degradation could be due to the interfacial degradation and ion accumulation at the contacts^11^ proved by the loss of FF. However, these effects could be reduced in the nanocomposite due to the presence of robust Maize in the mechanically fragile perovskite. Additionally, the Maize could slow the rate of perovskite decomposition by trapping the volatile MA cation and iodine species, limiting further degradation through diffusion and reactions that would occur in control devices (i.e., iodine reacting with the metal in contact).

Thermogravimetric analysis (TGA) was carried out to understand how the presence of the polymer around the perovskite grains influences the MAI sublimation, which is the main factor responsible for perovskite thermal degradation. TGA results of the perovskite material without and with Maize are reported in Figure 4b together with the derivative TGA (DTGA) results in Figure 4c. The three mass losses corresponding to MAPbI_3_ perovskite are observed: the first one attributed to the residual dimethyl sulfoxide (DMSO) evaporation^35,36^ (*T*_onset_ around 187.9 ± 5.9 °C), followed by the loss of organic component CH_3_NH_3_I (*T*_onset_ around 290.8 ± 9.3 °C) and second, the loss of HI and CH_3_NH_2_, as confirmed by the two peaks evidenced from the DTGA curve^37^ at around 319.8 and 334.0 °C, respectively. Finally, the weight loss with *T*_onset_ around 482.7 ± 16.7 °C is due to lead(II) iodide PbI sublimation.^35−37^

In the perovskite-containing Maize (red curve) the residual DMSO evaporation step was followed by a consequential loss step, as also evidenced by the peak at around 213 °C in the DTGA curve, that can be attributed to the loss of the polymer. Interestingly, a slight shift of the *T*_onset_ related to the MAI degradation weight loss up to 300.6 ± 1.2 °C was observed as well as the replacement of the MAPbI_3_ sample double peak in the DTGA with a peak at about 325.1 °C. The delay in the loss of MAI is also demonstrated by the slight 4% increase of the activation energy (*E*_a_) of MAI sublimation (Figure 4d) calculated from the slope of the linear fit of the data (based on eq 1) in the inset of Figure 4b, by applying the Broido model to the TGA curves of the samples.

The thermal characterization reinforced the hypothesis of the beneficial role of Maize surrounding the perovskite grains that prevented the physical loss of the MAI precursor, delaying the perovskite degradation and improving the thermal stability of the active material.

**Figure 5:**
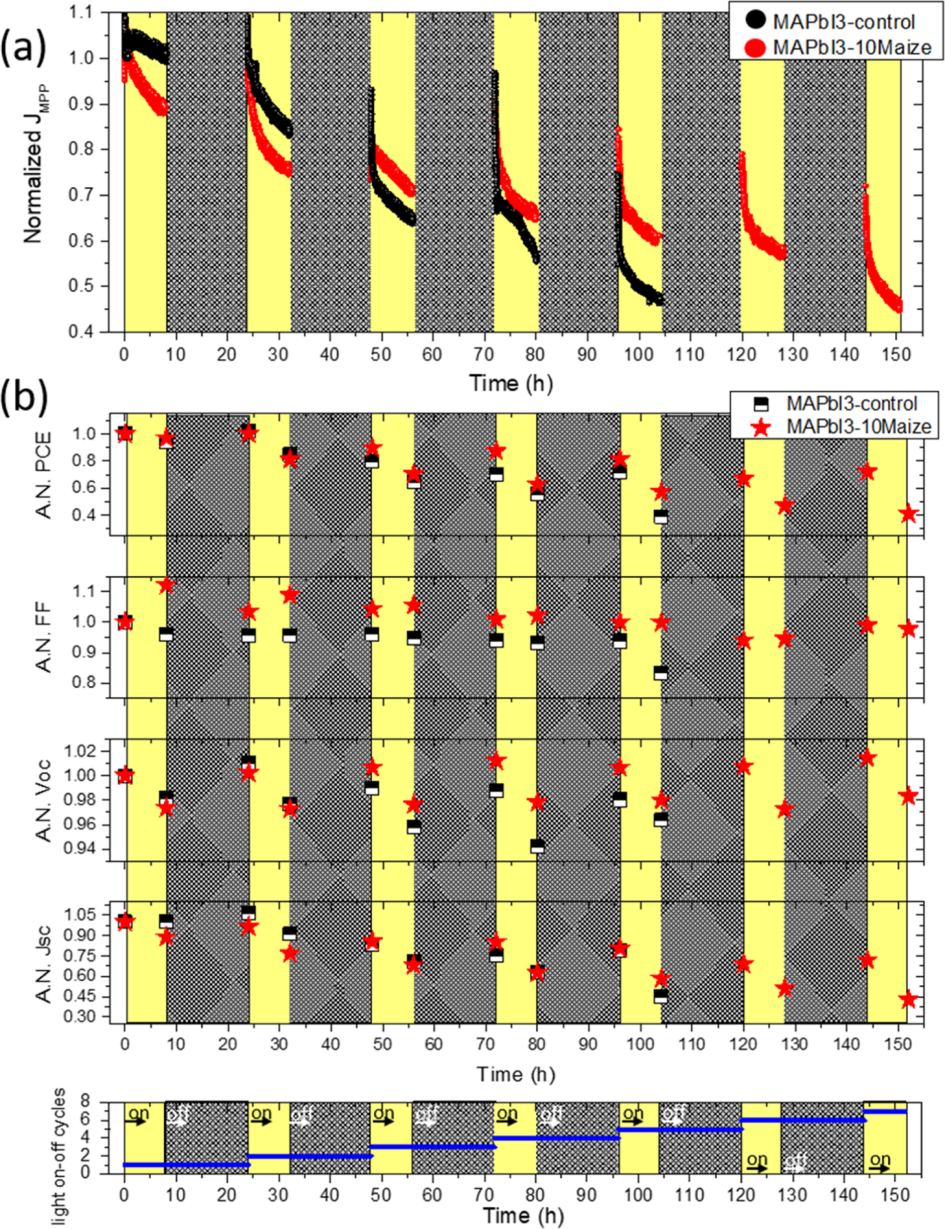
Normalized current density at the MPP (*J*_MPP_) extracted from continuous MPP tracking for MAPbI_3_-10Maize and MAPbI_3_-control measured under the ISOS-LC-1-like aging condition (a) and average normalized parameters of PCE, FF, *V*_OC_, and *J*_SC_ of MAPbI_3_-10Maize and MAPbI_3_-control measured under the ISOS-LC-1-like aging condition (b).

A further stability test of the developed nanocomposite was performed by applying realistic operational conditions to the unencapsulated device by tracking the current density at the maximum power point (MPP) under light cycling test (continuous irradiation for 8 h, followed by 16 h of dark), in ambient air (*T*: ∼ 25 °C; RH: ∼50%) to mimic the diurnal light–dark cycle in the presence of a moderate humidity content according to the ISOS-LC-1 protocol.^15^ In Figure 5a, the tracking of the normalized current density at the MPP (*J*_MPP_) was reported until the PCE of the devices dropped below 50% (Figure 5b). The *J*_MPP_ tracking of the devices under light cycling aging followed the typical double exponential decay of the maximum power output traces, irrespective of the perovskite composition.^38−40^ In detail, the initial rapid decay regime is representative of the intrinsic reversible performance losses,^38^ as also confirmed by the recovery *J*_MPP_ observed after leaving the devices in dark. However, the subsequent slower decay regime is due to several mechanisms involving the irreversible degradation of device components.^41,42^

It is interesting to note that during the first cycle, a slower *J*_MPP_ decay was observed for the MAPbI_3_-control-based device, mainly due to the stable *J*_sc_, while the *V*_oc_ and FF decreased by about 2 and 5%, respectively (Figure 5b). The MAPbI_3_-10Maize-based devices also showed a slight *J*_sc_ and *V*_oc_ reductions of about 10 and 2%, respectively; however, there was an increase in the FF of about 12%, an effect which further supports the improved stability of the interfacial contact between the perovskite and the hole-transport layer,^43^ potentially due to the denser morphology (visible in the cross-sectional SEM in Figure 2) and improved fracture energy that reduced the number of voids/degradation. This effect could be one of the key mechanisms for stability enhancement with starch incorporation.

Overall, the different aging response observed during the first cycle without and with Maize resulted in a similar PCE change in both perovskites. During the second cycle, a comparable *J*_MPP_ reduction rate was observed: the MAPbI_3_-control-based device started to show a *J*_sc_ reduction of about 10% compared to about 25% for MAPbI_3_-10Maize, which nevertheless showed an increase in the FF. From the third cycle, an inverted trend was observed, with a faster and higher *J*_MPP_ decay in the MAPbI_3_-control device caused by a rapid drop of *J*_sc_ and *V*_oc_ values, and after the fifth cycle, the FF decrease accelerated, eventually determining the decrease of PCE and *J*_MPP_ down to 40 and 45% of the initial values, respectively. Improved stability was observed instead in the presence of Maize, especially due to a stable FF and a slower decrease of both *V*_oc_ and *J*_sc_, showing 40% and 45% of the initial PCE and *J*_MPP_ value, respectively, after the seventh aging cycle. Compared with the standard MAPbI_3_-based device, the presence of Maize in perovskite also contributes to the improvement of the photostability of the device in the presence of humidity. A reversible degradation/recovery phenomenon was observed even in the presence of Maize, confirming that the reversible performance losses attributed to the slow cation migration are intrinsic to the perovskite material, regardless of the composition.^38^ Moreover, the presence of the biopolymer in the active layer of the device plays a beneficial role in retarding the perovskite degradation, possibly by improving the stability of the interfaces and by limiting the moisture–assisted ion migration and formation of defects,^44^ as demonstrated by the slower decrease of Voc and stable FF of the MAPbI_3_-10Maize-based device under operation.

## 3 Conclusions

In this work, we studied the influence of a long-chain starch polymer (Maize) additive on the thermomechanical properties and operational stability of MAPbI_3_.

We have previously demonstrated the multiple beneficial effects provided by the use of the Maize additive in perovskite film formation: (i) it interacts with MAI by hydrogen bonding, already in solution, forming a sol–gel network that delays and guides the crystallization process, allowing a better control of film formation; (ii) it modifies the rheology of perovskite precursor’s solution and guarantees the deposition of a compact, homogeneous, and high-quality film morphology in a single-step coating (antisolvent dripping-free); (iii) the hygroscopic nature of the long-chain of the Maize network stabilizes and protects the perovskite from decomposition in an ambient environment, while the plasticity of the polymer conferred enhanced resistance to bending stress.

The use of Maize increased the *G*_c_ of the film to above 5 J/m^2^, creating a nanocomposite that provided intrinsic reinforcement at perovskite grain boundaries. The uniform distribution of Maize throughout the film thickness at the grain boundaries enabled among the highest photovoltaic performance reported for fully organic p–i–n devices based on the MAPbI_3_-polymer active layer deposited in one step (dripping-free method), while enhancing the environmental stability. Compared with standard MAPbI_3_, the perovskite–Maize nanocomposite also contributed to improved photostability during diurnal cycles in the presence of moderate humidity and resistance to thermal cycling. The use of Maize is an effective strategy to improve several aspects concerning perovskite-based devices: from film processing, by allowing the deposition in a simple one-step dripping-free method, to the improvement of perovskite properties in terms of thermomechanical reliability, efficiency, and stability, even under accelerated operational conditions in an effort to achieve a commercially viable photovoltaic technology.

## 4 Experimental Section/Methods

### 4.1 Materials

Lead(II) iodide PbI, ultradry 99.999% (metal basis) was purchased from Alfa Aesar and MAI from Dyesol. Maize; DMSO anhydrous, 99.9%; chlorobenzene anhydrous, 99.8% (CB); 2-isopropanol (IPA); and BCP, 96% were purchased from Aldrich. Poly-TPD was purchased from Solaris Chem Inc. and PCBM was purchased from Nano-c. All the materials were used as received, without any further purification, with the exception of Maize which has been dried for 2 weeks at 60 °C before adding it to a perovskite precursor solution.

## 5 Methods

### 5.1 Starch–Perovskite Solution Preparation

The perovskite precursor solutions were prepared, as reported in previous work,^23^ by mixing MAI and PbI at an equimolar stoichiometric ratio (1:1) in DMSO with a precursor concentration of 30 wt %, followed by stirring at 80 °C for 30 min. After precursor solubilization, Maize was added to perovskite precursors in variable amounts (i.e., 0, 10, 15, and 20 wt %), followed by stirring at 80 °C for 5 h. All the solutions were prepared in a N_2_-filled glovebox. The ID of each sample and perovskite precursor/starch concentrations are reported in Table S1.

### 5.2 Starch–Perovskite Film Formation

The perovskite–Maize-based solutions were deposited by spin-coating at 9000 rpm for 20 s onto different substrates, followed by annealing at 100 °C for 30 min in a nitrogen atmosphere.

### 5.3 Device Fabrication

Glass ITO-coated substrates (Visiontek Systems Ltd.) were sequentially cleaned by ultrasonication in acetone and deionized water. After plasma treatment, a solution of poly-TPD (1.5 mg/ml in CB) was deposited on glass/ITO by spin-coating at 4000 rpm for 60 s, followed by annealing at 110 °C for 30 min in air and UV treatment in air for 30 min to improve the poly-TPD surface wettability.^45^ Subsequently, the device fabrication was completed in a N_2_-filled glovebox. The perovskite–starch formulations reported in Table S1 were spin-coated at 9000 rpm for 20 s on the poly-TPD film and then annealed at 100 °C for 30 min on a hot plate. PCBM (25 mg/mL in CB) and BCP (0.5 mg/mL in IPA) solutions were sequentially deposited on the active perovskite layer at 1000 rpm. for 60 s and at 5000 rpm for 20 s, respectively. Finally, Au cathodes were evaporated through a shadow mask in high vacuum. The active area of the device was 0.04 cm^2^.

For a reference device, the perovskite–starch film was replaced by a standard MAPbI_3_ perovskite, whose solution was prepared by dissolving (after stirring at room temperature for several hours) stoichiometric amounts of MAI, PbI, and DMSO (1:1:1) in dimethylformamide with a precursor concentration of 48 wt %. Then, the solution was deposited by spin-coating at 4000 rpm for 25 s, and the toluene was dripped onto the substrate after 15 s from the beginning of the process. The coated substrate was finally annealed on a hot plate at 100 °C for 10 min.

### 5.4 SEM–EDS Characterization

The samples were characterized by high-resolution (HR)SEM using a JEOL JSM-7500LA (JEOL, Tokyo, Japan) equipped with a cold field-emission gun, operating at 10 kV acceleration voltage. Back-scattered electrons were used in order to enhance the differences in the sample chemical composition. EDS (Oxford Instruments, X-Max, 80 mm^2^) was utilized to distinguish the presence and the distribution of Cs, I, O, and C. All experiments were done at 8 mm working distance, 10 kV acceleration voltage, and 15 sweep counts for each sample.

### 5.5 Device Characterization

Current–voltage characteristics of the devices were studied by using a Keithley 2400 Source Measure Unit and an Air Mass 1.5 Global (AM1.5G) solar simulator (Newport 91160A) under an irradiation intensity of 100 mW/cm^2^. The measurement was made setting a range of voltage from 1.2 to −0.2 V in the reverse mode.

The IPCE measurements were carried out on unencapsulated devices by using a Newport 140 W xenon lamp (66,920) power source with a Newport Cornerstone 260 monochromator, a Newport 2936-C power meter, and a Newport 71675_71580 photodiode.

### 5.6 *G*_c_ Measurements

In order to measure *G*_c_ with a DCB test, a setup comprising a load cell and an actuator was used to control the displacement of the specimen (delaminator testing system) and to generate a load versus displacement curve. A uniaxial load was applied to each glass beam normal to the films on one end of the test structure. Displacement of the beams continued until the specimen underwent critical fracture. The specimen was loaded in tension with a displacement rate of 1 μm s^–1^ until reaching the critical load (*P*_c_)—the value for which the load dropped at a given displacement—before unloading slightly to calculate the compliance of the specimen, *C* = dΔ/d*P*. The specimens were then loaded again to *P*_c_ and the process was repeated iteratively until the crack length reached the end of the specimen. The compliance relation was used to extract *a* during the test using the previously defined specimen dimensions and the plane-strain elastic modulus, ***E***′
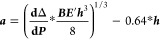
1

The elegance of the DCB test is that no further information is required once the load–displacement curve is obtained in order to calculate *G*_c_. The fundamental definition of the strain-energy release rate is derived from the change in potential energy from a loading system and is given by eq 2
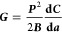
2

This result is independent of the loading configuration and therefore can directly be applied to the DCB geometry assuming the compliance relation is known. Beam mechanics can be used—the details of which are left to the engineers—in order to derive the moment of inertia (*I*) and the relationship between the displacement and the moment. The result is given as eq 3
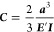
3

Substituting eq 2 into eq 3 in the critical condition yields
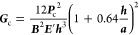
4where the term in parentheses is known as the Kanninen correction^46^ which accounts for the boundaries of the beams. Note that a *G*_c_ value was calculated for each critical load and averaged in order to obtain multiple data points per specimen.

### 5.7 Thermogravimetric Analysis

TGA of the perovskite samples without and with 10 wt % of Maize was carried out using a TA Instruments SDT Q600. About 10 μL of precursor solution was put in an alumina holder and annealed at 100 °C for 1 h under a nitrogen atmosphere to allow the perovskite formation after solvent evaporation. Subsequently, the samples were heated through a dynamic ramp up to 700 °C at a heating rate of 10 °C/min in order to evaluate the thermal stability of the samples. Three measurements were performed on each sample. The onset temperature, *T*_onset_, was calculated by extrapolating the intersection point between the tangent lines to the curve at the beginning of the weight loss.

A further kinetic analysis of MAI thermal decomposition was carried out by applying the Broido method to the respective weight loss step based on eq 5
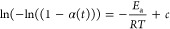
5where

- (t) is the degree of reaction of the sample component during degradation at a given time *t*, defined as

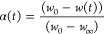
where *w*_0_, *w*(t), and *w*_∞_ are the weight of the sample before degradation, at a certain time t during degradation, and after degradation, respectively.

- *E*_a_ is the activation energy,
- *R* is the gas constant (8,31 J/mol K).

By applying this method for TGA of the samples, the slope of the straight line observed between ln(−ln(1 – α(*t*))) and 1/T is obtained, represented as −*E*_a_/*R*. Therefore, by interpolating with a linear fit, the activation energy of the degradation process without and with Maize can be calculated.

### 5.8 Durability Test

#### 5.8.1 Light Aging Test

Light aging tests were performed by tracking *J*_MPP_ of the unencapsulated devices under a light cycling test (continuous irradiation for 8 h, followed by 16 h of dark), in ambient air (temperature: ∼25 °C; moisture content: ∼50%) to mimic the diurnal light–dark cycle in the presence of a moderate humidity content according to the ISOS-LC-1 protocol.

#### 5.8.2 Thermal Aging Test

Thermal aging tests were performed by placing the solar cells at 85 °C on a hot plate in a N_2_-filled environment and in the dark for several hours (details are reported in Figure 4), alternating about 8 h at room temperature between each high-temperature step.

#### 5.8.3 PL Measurements

TRPL was performed using a Horiba Fluorolog fluorimeter. Perovskite films on poly-TPD/glass were excited with a 635 nm laser to give time-correlated single-photon counting lifetime measurements.

#### 5.8.4 X-ray Photoelectron Spectroscopy

XPS was performed with a PHI VersaProbe3 system using a monochromatized Al(*K*_a_) source at 1486 eV. The elemental surface composition was characterized by high-resolution binding energy measurements of chemical spectra under high vacuum.

## Supporting Information Available

Chemical structure of Maize, sample ID and composition, *J*–*V* curves measured in reverse and forward scan modes for MAPbI_3_-10Maize, and IPCE spectra for MAPbI_3_-10Maize- and MAPbI_3_-control-based devices (PDF)
