# Supplementary material for: Robust, High-Performing Maize–Perovskite-Based Solar Cells with Improved Stability
Source: ACS Appl Energy Mater. 2021 Sep 27;4(10):11194–203. doi: 10.1021/acsaem.1c02058 (PMC9342243; doi:10.1021/acsaem.1c02058)
Supplement: Supplementary file 1 — ae1c02058_si_001.pdf [file ae1c02058_si_001.pdf]

Supporting Information

Robust, high-performing maize-perovskite based solar cells with improved stability

"Antonella Giuri[§,#], Nicholas Rolston[†,#], Silvia Colella[‡] , Andrea Listorti[‖], Carola Esposito Corcione[§,⊥], Hannah Elmaraghi[†], Simone Lauciello[ᶲ], Reinhold H. Dauskardt[†,] and Aurora Rizzo [§,*]*

# Equally contributed to this work

*Corresponding authors: rhd@stanford.edu; aurora.rizzo@nanotec.cnr.it

Dr. Antonella Giuri
[§]CNR NANOTEC, Institute of Nanotechnology, Via Monteroni, 73100 Lecce, Italy

Dr. Nicholas Rolston, Hannah Elmaraghi, Prof. Reinhold H. Dauskardt

[†]Department of Materials Science and Engineering, Stanford University, Stanford, CA, USA

Dr. Silvia Colella

[‡] CNR NANOTEC - Istituto di Nanotecnologia, Dipartimento di Chimica, Universita` degli Studi di Bari Aldo Moro, Via Orabona 4, 70126 Bari, Italy

Dr. Andrea Listorti

[‖] Dipartimento di Chimica, Universita` degli Studi di Bari Aldo Moro, Via Orabona 4, 70126 Bari, Italy

Dr. *Carola Esposito Corcione*

[§]CNR NANOTEC, Institute of Nanotechnology, Via Monteroni, 73100 Lecce, Italy

[⊥]Dipartimento di Ingegneria dell'Innovazione, Università del Salento, via per Monteroni, km 1, 73100 Lecce, Italy

Simone Lauciello

[ᶲ]Electron Microscopy Facility, Istituto Italiano di Tecnologia, via Morego 30, Genova (Italia)

Dr. Aurora Rizzo (corresponding author)

[§]CNR NANOTEC, Institute of Nanotechnology, Via Monteroni, 73100 Lecce, Italy

E-mail: aurora.rizzo@nanotec.cnr.it"

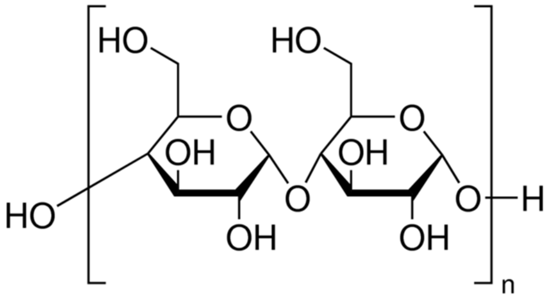

Figure S1. Chemical structure of Maize Corn starch

Table S1. Sample ID and composition

Sample ID	Maize/$MAPbI_3$ wt%
$MAPbI_3$	0
$MAPbI_3$-10Maize	10
$MAPbI_3$-15Maize	15
$MAPbI_3$-20Maize	20

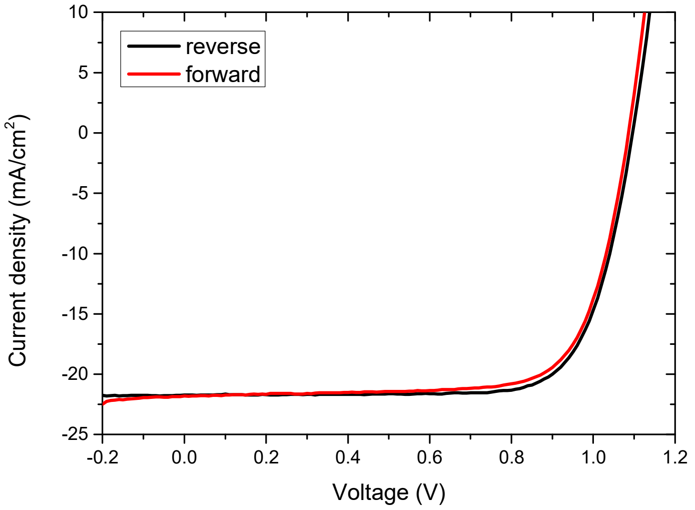

Figure S2. JV curves measured in reverse and forward scan mode for $MAPbI_3$-10Maize

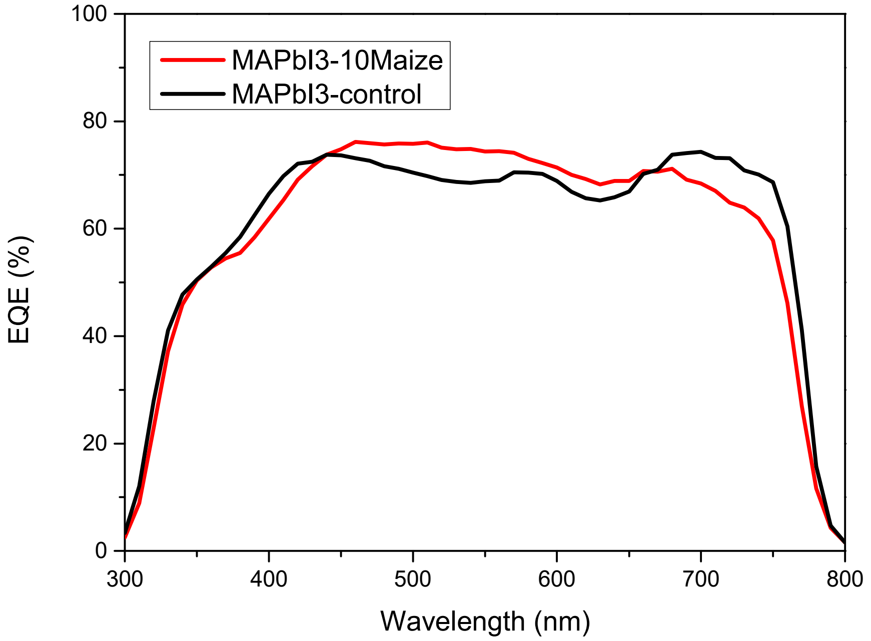

Figure S3. IPCE spectra for $MAPbI_3$-10Maize and $MAPbI_3$-control based devices.
